# Supplementary material for: Iron and Copper Doped Zinc Oxide Nanopowders as a Sensitizer of Industrial Energetic Materials to Visible Laser Radiation
Source: Nanomaterials (Basel). 2022 Nov 24;12(23):4176. doi: 10.3390/nano12234176 (PMC9736272; doi:10.3390/nano12234176)
Supplement: Supplementary file 1 [file nanomaterials-12-04176-s001.zip › nanomaterials-2050642-supplementary.pdf]

Supplementary Information

Energy distributions across a laser beam of 450 nm diode and Nd:YAG laser are shown in fig. S1 and fig. S2 respectively. Solid black lines are plotted using the energies of the laser beam parts passing through the circle diaphragm. A set of 5 diaphragms with different diameters was used. Color dashed lines are the approximation of obtained data by Gaussian. The unfocused laser beam print of the IR diode and its energy distribution is shown in fig. S3.

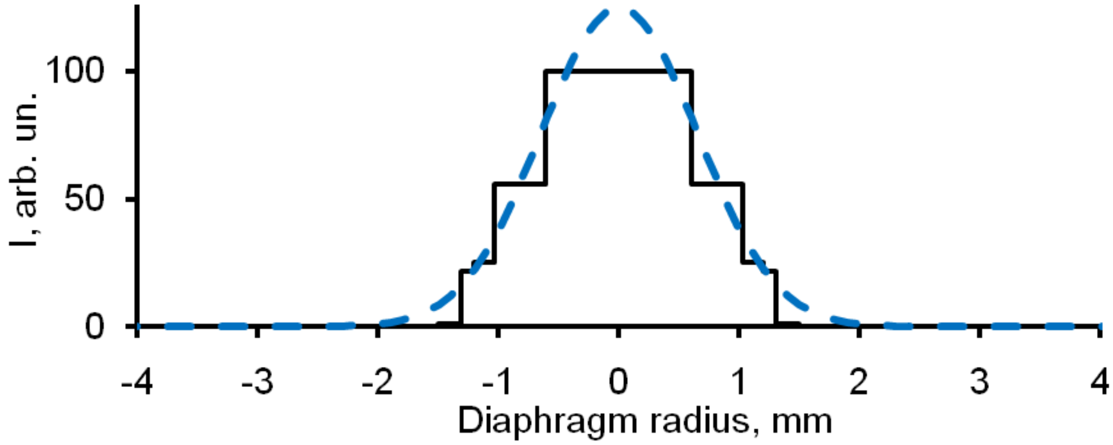

Figure S1 Energy distributions across a laser beam of 450 nm diode.

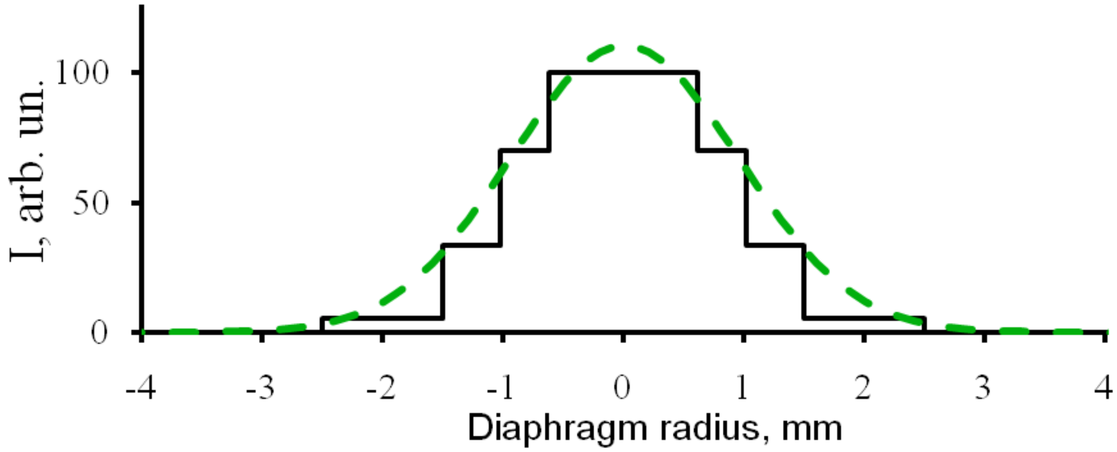

Figure S2 Energy distributions across a laser beam of Nd:YAG (second harmonic, 532 nm).

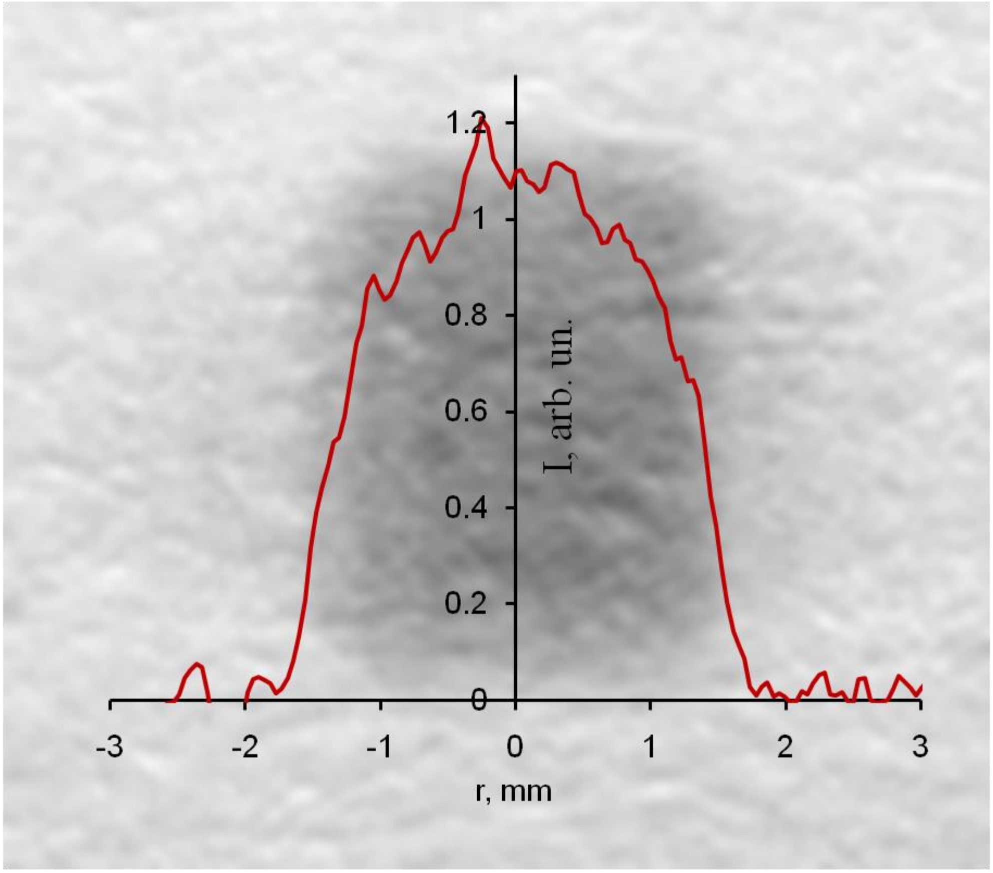

Figure S3 Laser beam print and energy distribution of the 808 nm diode.
